# Strength and ductility with {101̄1} — {101̄2} double twinning in a magnesium alloy

**DOI:** 10.1038/ncomms11068

**Published:** 2016-04-04

**Authors:** M. Lentz, M. Risse, N. Schaefer, W. Reimers, I. J. Beyerlein

**Affiliations:** 1 Technische Universität Berlin, Institut für Werkstoffwissenschaften und -technologien, Metallische Werkstoffe, Ernst-Reuter-Platz 1, Berlin 10587, Germany; 2 Helmholtz-Zentrum Berlin für Materialien und Energien GmbH, Institute Nano-architectures for Energy Conversion (EE-IN), Hahn-Meitner-Platz 1, Berlin 14109, Germany; 3 Los Alamos National Laboratory, Theoretical Division, Los Alamos, New Mexico 87545, USA

## Abstract

Based on their high specific strength and stiffness, magnesium alloys are attractive for lightweight applications in aerospace and transportation, where weight saving is crucial for the reduction of carbon dioxide emissions. Unfortunately, the ductility of magnesium alloys is usually limited. It is thought that one reason for the lack of ductility is that the development of 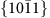
—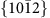 double twins (DTW) cause premature failure of magnesium alloys. Here we show with a magnesium alloy containing 4 wt% lithium, that the same impressively large compression failure strains can be achieved with DTWs as without. The DTWs form stably across the microstructure and continuously throughout straining, forming three-dimensional intra-granular networks, a potential strengthening mechanism. We rationalize that relatively easier <c+a> slip characteristic of this alloy plastically relaxed the localized stress concentrations that DTWs can generate. This result may provide key insight and an alternative perspective towards designing formable and strong magnesium alloys.

## Abstract (web summary)

Double twins are thought to lead to premature failure of magnesium alloys. Here, the authors show that a magnesium-lithium alloy achieves the same compression failure strains with double twins as without, potentially generating strengthening through intra-granular networks of double twins.

## Graphical abstract

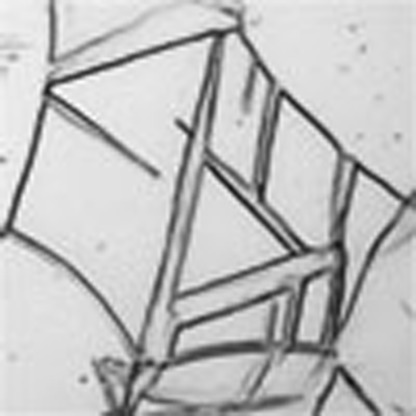

Magnesium alloys have attracted much attention as candidate metals for structural applications, particularly when properties such as high strength and lightweightness are highly desired. Although Mg possesses a high specific strength and Young’s modulus, it is not ductile and exhibits pronounced mechanical anisotropy, posing a bottleneck to its widespread use. It is thought that one of the reasons for the lack of ductility is the development of a certain kind of deformation twin, the double twin (DTW).

The most common twin modes in Mg alloys are 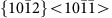 tension twinning (TTW-ing) and 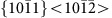 compression twinning (CTW-ing)^1^,^2^,^3^,^4^,^5^,^6^,^7^,^8^,^9^,^10^,^11^,^12^. CTWs and TTWs are single twins, the former activated to accommodate contraction strains and the latter, extension strains along the *c* axis^13^. Experimental studies on various Mg alloys have found that TTWs can grow thick, and even overtake the grain, and generally reach profuse levels without failing the material^9^,^10^,^11^,^14^,^15^. These twins can improve ductility, but lower the strength^1^,^3^,^16^. CTWs, on the other hand, remain fine and nucleate a second TTW inside their domain^4^,^9^,^10^,^17^,^18^,^19^. This internal TTW expands, overtaking the fine CTW lamella. The result is a 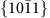
—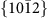 DTW.

In several studies, DTWs have been correlated to void and crack formation and flow localization in the vicinity of its boundaries^4^,^16^,^17^,^18^,^20^. On this basis, research has aimed to reduce or suppress CTW and DTW development with alloying or grain size reduction^4^,^10^,^16^,^21^.

These prior works studied pure Mg or Mg alloys in which <c+a> slip was difficult relative to <a> basal slip (ratios in the range of 12–15)^3^,^22^. In these materials, plastic relaxation of the stress concentrations that occur at DTW boundaries or twin tips is limited. Consequently, cracks and voids form in the vicinity of the DTWs, inducing failure. However, it is not the deformation twins themselves that cause failure but the lack of plastic relaxation mechanisms. If more plastic deformation modes were easily available to relieve stress concentrations, then crack and void formation could be hindered at the DTWs and other defects or discontinuities. In this case, it would not be necessary to eliminate DTW-ing and in fact, doing so could miss a potentially important strengthening mechanism.

In this work, we investigate the effect of DTW-ing on the compression failure strain in an Mg-4wt%Li alloy, in which activation thresholds for <c+a> slip are closer to <a> slip (ranging from 8 to 10)^11^,^23^. The ease of <c+a> pyramidal slip, which has been firmly established through transmission electron microscopy (TEM)-based studies by various authors^24^,^25^,^26^, is postulated here to improve the formability facilitating *c* axis deformation, fulfilling the Taylor criterion and providing an effective plastic relaxation mechanism. Here, we show that the onset and even profuse growth of CTWs and DTWs do not lead to failure. The same alloy, one a fine-grained (FG) material that deforms with negligible contraction twinning and the other a coarse-grained (CG) material with ample CTW-ing and DTW-ing, achieved similar compression strains without failure under similar levels and states of strain and stress. Most interestingly, we reveal that many CTWs and DTWs can form within the same grain. They multiply by remaining relatively thin, but forming three-dimensional (3D) intra-granular networks, which refine the grain size and could lead to strengthening. A combined in-grain misorientation axes (IGMA) analysis and crystal plasticity calculations suggests that this seemingly uncharacteristic behaviour results from the enhanced activity of <c+a> pyramidal slip in the present Mg alloy relative to those previously studied. These findings offer insight that could help address the challenge of achieving formable and strong magnesium alloys.

## Results

### Processing and mechanical properties

**Figure 1:**
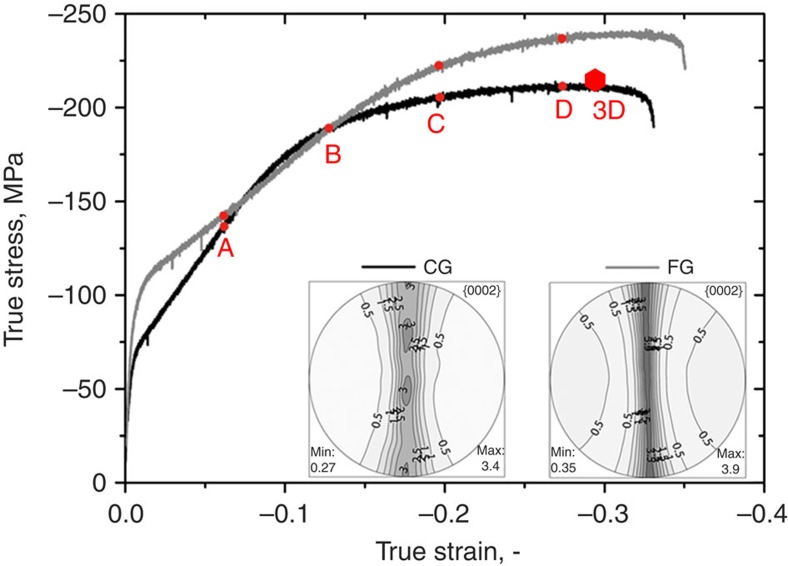
Compression flow curves with load direction being radial. The compression tests were repeated at least five times. Insets represent the texture using {0002} pole figures (⊥ED). The texture of the materials is almost equivalent. Hence, texture effects are largely omitted in this study. According to the Hall–Petch relation, the FG material features higher yield strength; however, the CG material exhibits more pronounced work-hardening initially. (A–D) Strain levels for EBSD analysis, while the hexagon labels the strain level for serial sectioning analysis. ED, extrusion direction.

The starting material is an Mg-4wt%Li alloy, purchased from the Leibniz Universität Hannover (Institut für Werkstoffkunde) in as-cast round billets. To refine the grain size, the billets were extruded into rods at the Extrusion Research and Development Center (FZS—TU Berlin) using an indirect extrusion process at a product speed of 1.7 m min^−1^. The samples were then cut and polished using standard processes for subsequent characterization of the grain structure, size and shape, via optical microscopy (see Methods). The CG material extruded with a ratio of 41:1, at a billet temperature of 300 °C, and air cooling had a uniform, average grain size of 23 μm. Another sample (FG) extruded with a larger ratio 71:1, lower temperature 200 °C, and water cooling had a finer, uniform average grain size of 5 μm. The CG and FG samples were also prepared for orientation mapping by electron backscatter diffraction (EBSD). Using a 0.5 μm step size, the EBSD analysis and optical microscopy showed that initially the grains were free of twins above a thickness of ∼300 nm. Although the materials feature different grain sizes, both fully recrystallized dynamically during the hot extrusion process resulting in very similar textures, which differ only in sharpness (c.f. Fig. 1 inset, Supplementary Fig. 1 and Supplementary Note 1), and hence low dislocation densities in the as-extruded condition can be reasonably presumed. Due to the structure of the hexagonal close packed (HCP) crystal, the activation of slip and twinning can be highly dependent on texture^5^,^7^,^11^,^22^,^23^,^27^,^28^,^29^. On the basis of these preliminary characterizations and elastic–plastic self-consistent (EPSC) simulations, we anticipate that changes in the deformation twinning propensity between the CG and FG samples will arise because of the differences in their grain size, and not in their starting texture, initial dislocation density or initial twin fraction.

Both materials were machined into specimens (Ø=7.5 mm, l_0_=15 mm (3D serial section); Ø=5 mm, *l*_0_=10 mm (flow curves and EBSD)) for compression testing normal to the extrusion direction. Figure 1 compares the deformation response of the CG and FG samples, while Supplementary Fig. 2 displays a schematic of the barrelling of the samples. The FG sample has a higher yield and failure stress and lower hardening rate than those of the CG sample. In Supplementary Fig. 3, we show, using the hardening parameters from ref. 11, that neither differences in texture sharpness nor changes of the dislocation density, within an expected range, create the different strain hardening behaviour. An important similarity, however, is that both materials exhibit a high compression failure strains of 33% (CG) and 35% (FG), larger than that achieved by pure Mg (ref. 27) and other alloys such as AZ31 (refs 3, 16, 30, 31) and ZK60 (ref. 32). While these large compression failure strains are consistent with prior reports on Mg–Li alloys, which were attributed to an enhancement of <c+a> pyramidal slip^23^,^24^,^25^,^26^,^31^, it is interesting that they are similar between two samples differing significantly and predominantly in average grain size.

### Microstructure evolution during compression

**Figure 2:**
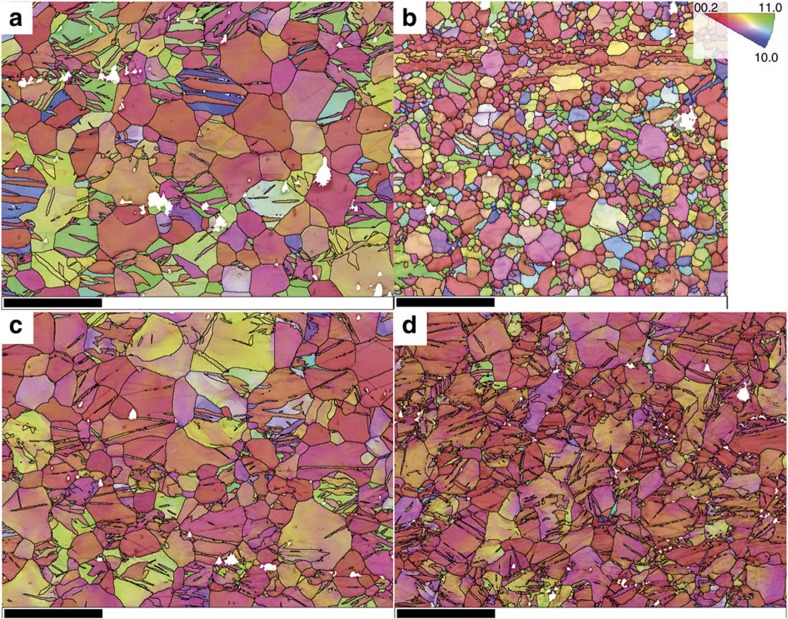
EBSD maps representing the orientation of the grains through inverse pole figure colour-coding. (**a**) CG (ε_E_=6%), (**b**) FG (ε_E_=6%), (**c**) CG (ε_E_=12%) and (**d**) CG (ε_E_=24%). TTW-ing is more pronounced in the CG samples. Within the strain range from 12 to 24% DTWs formed in the CG material in contrast to the FG material. As the EBSD analysis was applied to reveal the orientation and misorientation relations, only 1 to 2 EBSD data sets were collected for each sample. In addition microstructural evolution was confirmed by numerous optical micrographs containing several hundreds of grains. Scale bars (**a**,**c**,**d**), 100 μm. Scale bar (**b**), 50 μm.

**Figure 3:**
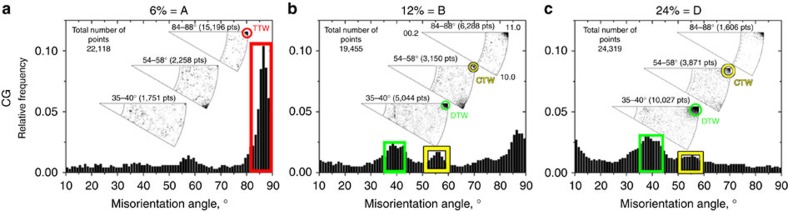
MDFs of the CG material at different strains. At *ε*_E_=6%, TTW-ing is predominant, while at *ε*_E_=12% TTW-ing is almost exhausted and first CTWs and DTWs have formed. (**a**) *ε*_E_ 6% (Point A in Fig. 1), (**b**) *ε*_E_ 12% (Point B in Fig. 1). (**c**) *ε*_E_ 24% (Point D in Fig. 1). During further loading, DTWs become the dominant microstructural feature. The MDFs were computed from the EBSD data sets represented in Fig. 2. The characteristic misorientation axes and angles have been labelled using red (TTW), yellow (CTW) and green (DTW) boxes and circles.

At points A and B in their constitutive response (Fig. 1), the stress and strain levels of the two materials are the same, an ideal situation in which to compare their microstructure and underlying twin fractions. At these two points, EBSD inverse pole figure maps (Fig. 2), EBSD band contrast maps highlighting twin boundaries (Supplementary Figs 4 and 5; TTW=red, CTW =yellow and type 1 DTWs=green) and their corresponding misorientation distribution functions (MDFs; Figs 3 and Supplementary Fig. 6) are used to identify twins and their variants in deformed samples. In both samples at point A (6% engineering strain ε_E_), 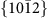, TTWs have formed in the grains and overtaken them. These can be recognized by the reorientation of the *c* axis by ∼86° about an 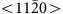 axis. However, fewer grains have been overcome by TTWs in the FG sample than in the CG sample, a mild grain size effect consistent with prior reports^10^,^11^,^16^,^30^,^33^,^34^,^35^,^36^ (Fig. 2 and Supplementary Figs 4 and 5). In the Supplementary Information, we calculate the TTW volume fraction (Supplementary Table 1 and Supplementary Note 2) and do not observe major differences between the two materials within the considerable margin of error. Yet both materials contain a sufficiently large number of grains with the *c* axis nearly parallel to the compression direction. Consequently, there is plenty of volume for potentially detrimental CTWs and DTWs to form with continued straining.

A combination of EBSD orientation maps (using inverse pole figure colour-coding), band contrast maps highlighting twin boundaries and misorientation distributions is used to reveal the possible progression of twinning with further applied straining from *ε*_E_=6–12% (Supplementary Figs 4 and 5). Supplementary Fig. 4 shows the band contrast maps for the CG material at 12% engineering strain. Here twins are distinguished by their twin-matrix orientation relationship, with red as TTW, yellow as CTW and green as DTW. When a CTW transitions to a DTW by developing an internal 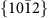 twin, it produces a misorientation angle of 37.5° about an 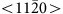 axis. Other DTW variants are possible (exhibiting other misorientation angles^37^,^38^, but these are found much less frequently). In Supplementary Fig. 4, we see that a high volume fraction of CTWs and especially DTWs has formed between applied engineering strains of 6–12%. Both the CTWs and DTWs are fine and needle-like with an average thickness of ∼3 μm. In Fig. 3, we present the MDFs for another quantitative assessment. A CTW exhibits a characteristic misorientation angle of ∼56°, about the 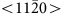 axis. Unlike at 6% engineering strain, a strong peak at 38° in the MDF has emerged, meaning that many CTWs have formed and already transitioned to DTWs. The weaker peak at 56° means that very few CTWs are found and these may have not yet transitioned to DTWs.

Repeating the same analysis for the FG material at 12% engineering strain (see Supplementary Fig. 6), we see that CTW-ing and DTW-ing are largely suppressed in the FG material, although stress and strain are very similar for the FG and CG materials (cf. Fig. 1). It is evident from the band contrast map that the FG material develops a very low fraction of CTWs and DTWs even at high strains (Supplementary Figs 5 and 6). Microstructural characterization of the FG material deformed to the largest strain level *ε*_E_=24% also finds very small fractions of CTWs or DTWs (Supplementary Figs 5 and 6). Therefore, even at larger strains and stresses, CTWs and DTWs did not form in noticeable amounts in the FG material. Clearly, the difference in average grain size was sufficient to hinder the onset of CTWs and DTWs in the FG material yet activate it profusely in the CG material.

We repeat the microstructural characterizations on the CG material deformed to much larger engineering strains of 18 and 24%. With further loading from *ε*_E_=12–24%, DTWs predominant the CG microstructure. The MDFs at *ε*_E_=12–24% in Fig. 3 show pronounced maxima at a misorientation angle of 38° indicating that DTWs dominated the microstructure. We observe from the corresponding band contrast map in Supplementary Fig. 4 that grains contain mostly DTWs (green boundaries) and far fewer CTWs (yellow boundaries), indicating that the CTW–DTW transition occurred in most cases. Increasing the strain from *ε*_E_=12–18% leads to the formation of new CTWs, which in turn are rapidly overtaken by DTWs. The CTWs are not necessarily parallel to the previously formed CTWs and consequently intersections of different twin lamellae occur. Increasing strain results in the formation of 3D DTW subgrain networks, which finely subdivide the parent grain. To summarize, the analysis demonstrates that the Mg alloy not only experienced ∼15–20% more strain after the onset of DTWs but also sustained a continued increase in the volume fraction of CTWs and DTWs. Therefore, the onset of CTWs and DTWs did not result in immediate failure.

### Three-dimensional arrangement of CTW/DTW networks

**Figure 4:**
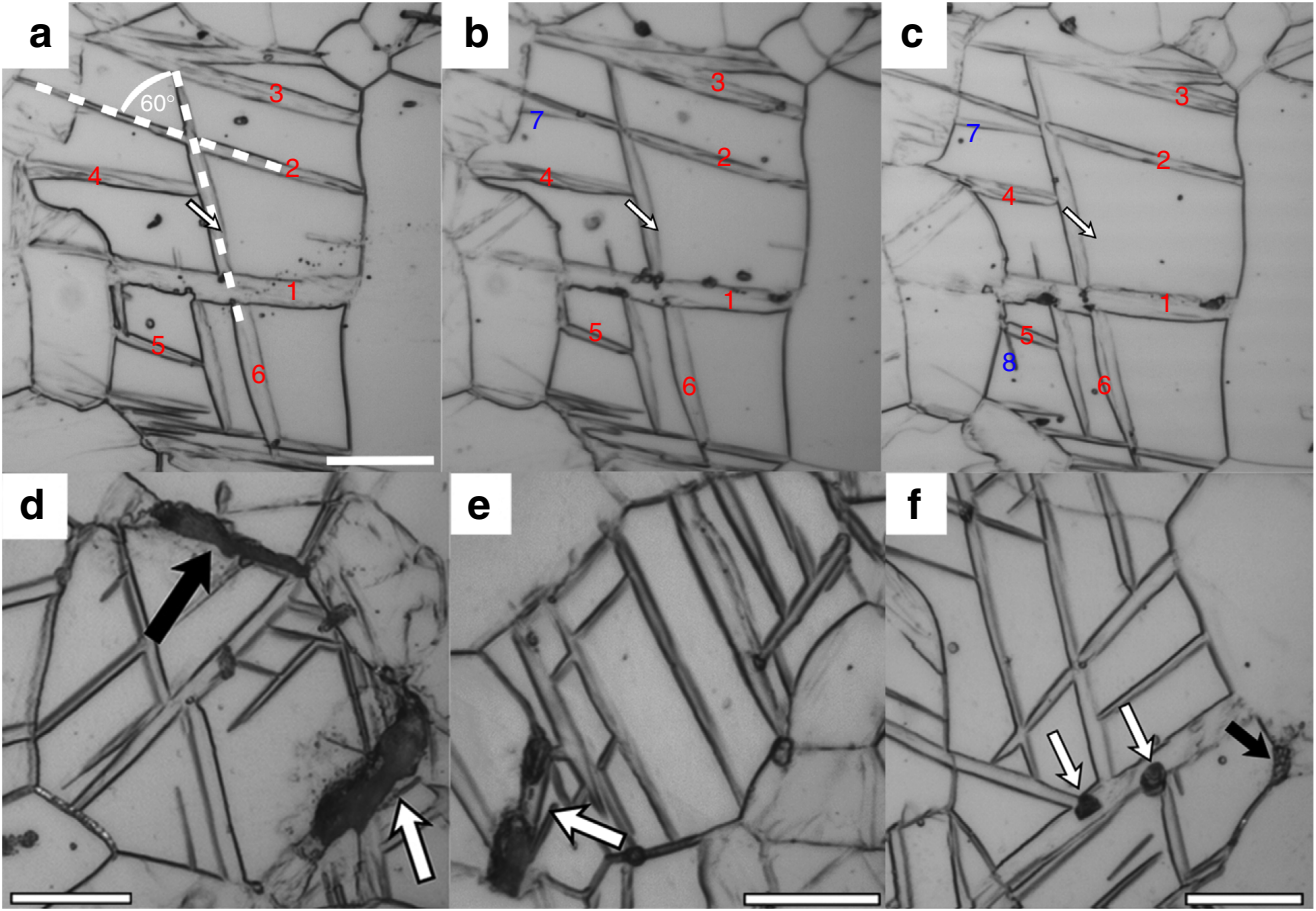
3D structure of contraction twin networks. Micrographs illustrating the 3D structure of the contraction twin network (**a**–**c**) and sites of crack and void formation (**d**–**f**). The 3D contraction twin network is generated through the formation of several CTW variants, which are subsequently overtaken by internal TTWs. Crack formation occurs at grain boundaries (black arrows), contraction twins or twin intersections (white arrows). Numbers given in (**a**–**c**) label specific contraction twin lamellae to facilitate their identification after serial sectioning. Red numbers denote twins, which were observed in all sections, while blue numbers denote twins, which were not observed initially, but during consecutive sectioning. Hence, the latter twins do not cross the entire grain. The 3D structure as well as the crack and void formation were evaluated in 10 grains using serial sectioning, while intersecting contraction twins and contraction twin networks were observed in hundreds of grains using conventional optical microscopy. Scale bar, 20 μm.

With this material, we have the opportunity to study the twin patterns that develop when an unusually high density of CTW and DTW lamellae can be generated. To obtain a 3D picture, we performed serial sectioning and optical microscopy on CG samples compressed to ∼29.5% strain, which still have not yet failed. Figure 4a–c (and Supplementary Fig. 7) display the complex 3D arrangement of a representative CTW/DTW network, among which there were many. Here, the white arrow is used as a reference point facilitating the tracking of twin boundary shifts. Several CTWs are labelled with red numbers to facilitate the relocation of the twins in each of the sections. Blue numbered CTWs were not visible initially and appeared during sectioning. The parent CTWs of the DTWs formed along different 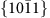 twin planes and hence, correspond to different twin variants. For example, twins 2, 3 and 5 feature similar alignment and shift towards the top of the image during sectioning and hence likely correspond to the same CTW variant (for example, variant C in Supplementary Fig. 8). However, twin 6 shifts towards the right side of the image and features a ∼60° angle to twins 2, 3 and 5. Assuming that the grain is aligned with its *c* axis perpendicular to the metallographic sections, the twin variants that do not share a 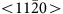 zone axis will be misoriented by 60° between the major axis of the two twin lamellae (cf. Fig. 4a–c and Supplementary Fig. 8), and hence twin 6 was generated through the activation of another CTW variant (for example, variant B in Supplementary Fig. 8). The twins 1 and 4 are similarly aligned suggesting that these twins share a 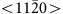 zone axis (variants A and C in Supplementary Fig. 8) and are the same variant. They are a different variant than the others. Twins 7 and 8 are not visible in the initial sections and appear after several polishing steps. These twins did not cross the entire parent grain. Presence of these twin–twin junctions and twin tips apparently did not result in immediate failure.

From Fig. 4a–c it is evident that the parent grain is successively subdivided into various subgrains by the generated DTW network, which evolves at strains >12%. The generated DTW boundaries introduce barriers for gliding dislocations and could potentially increase the strength through grain refinement. Here, it should be noted that the difference in work-hardening between the FG and the CG materials at strains <12% cannot be attributed to DTW formation based on the negligible amount of DTWs within this strain range. The strengthening is expected to depend on the slip mode and the twin^39^,^40^,^41^,^42^. This is particularly relevant in case of DTW boundaries. As DTWs are generated through the formation of a CTW, which is subsequently overtaken by an internal TTW, the DTW-matrix interface adopts an unconventional ‘twin plane’. On the basis of Kikuchi pattern analysis in TEM, it has been found that the boundary plane corresponds to an ∼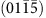 plane. Further high-resolution TEM has shown that this special plane is realized on the atomic scale by very thin residual CTWs along the DTW-matrix interface^9^. The complexity of such an interface suggests that DTW boundaries could be effective barriers hindering dislocation motion. Similarly, previous studies conducting constitutive modelling predicted an important strengthening effect of CTW-ing reducing the mean free path of gliding dislocations^43^,^44^. Therefore, we suggest that profuse DTW-ing could provide an additional way of strengthening Mg alloys through the generation of 3D networks, which subdivided and refined the parent microstructure. Nevertheless, more detailed experimental and simulation based studies are required to provide a comprehensive analysis of the DTW network effect on strength.

### Crack and void formation

In addition, we analysed various grains in the CG material (at ∼29.5% strain; hexagon in Fig. 1) with respect to flow localization, voids and cracks using serial sectioning (Fig. 4d–f), Supplementary Figs 9 and 10). Figure 4d–f illustrates grains, where void and crack formation was observed. Generally, we observed cracks and voids at both DTW boundaries (white arrows) and general grain boundaries (black arrows). In Fig. 4d, a grain boundary crack was observed. Through consecutive sectioning (Supplementary Fig. 9) we find that this crack was deflected by a CTW/DTW. Another crack is visible at the top of the image, which evolves along a grain boundary. However, Fig. 4e and Supplementary Fig. 10 demonstrate a tiny crack, which is clearly associated to a DTW. In addition we observed void and crack formation at a twin intersection (white arrows in Fig. 4f). The observed cracks do not feature sharp crack tips regardless of their origin. The statistics are too limited to determine whether grain boundary or twin-induced cracks occur more often. Nonetheless considering all the above observations it appears that cracks are just as likely to originate at grain boundaries as twin boundaries. Most importantly, despite the large number of fine CTWs and DTWs, twin-grain boundary junctions, and twin–twin junctions within the twin networks, local cracking neither initiates preferentially at these points nor when these types of defects are first produced.

This finding is very surprising as previous studies^4^,^17^,^18^,^20^ on DTW-ing using optical microscopy, scanning electron microscopy (including the evaluation of local deformation using grids scribed with focused ion beam) and TEM provide concurrent evidence of flow localization, void formation and crack formation associated to DTWs in pure Mg as well as in ZK60 and AZ31 alloys.

Explanations for DTW-induced failure have been based on preference for basal slip within the narrow DTW^4^,^16^,^17^,^18^,^20^. The parent grain has its *c* axis (nearly) parallel to the compression axis, and is well oriented for CTW-ing and <c+a> pyramidal slip, where CTW-ing usually reported to be harder than <c+a> slip^27^,^44^. The DTW domain, with the *c* axis reoriented 38° from the compression axis, is well oriented for the easier basal <a> slip. With the orientation of the fine DTW lamellae much more favorable for the activation of easy <a>-basal slip than the parent grain^4^,^17^,^18^, stress concentrations develop in the parent grain at the ends of the DTW in the untwinned region.

## Discussion

In the previous section we provide evidence that the onset of DTW-ing cannot be associated with immediate cracking (or void formation) and thus, the conventional explanation given in the prior paragraph is not complete. The reason is that it is based on the orientation relationship of the DTW to parent and hence applies to all HCP metals. It certainly applies to the present material. TEM and high-resolution TEM analyses have shown high basal slip activity within the DTW, the CTW and the surrounding grain^9^ and thus, this phenomenon alone is not the sole reason why DTW-ing led to failure in these other Mg alloys. Furthermore, the narrow morphology of those CTWs and DTWs in other studies is also not the reason. Here these twins are also just as fine. Furthermore, as we have shown, when permitted to grow to high fractions, many more ‘troublesome’ features, such as terminating twin tips and twin–twin junctions, are generated that supposedly could trigger localization, voids or cracks. These regions begin to become prevalent around 12% strain, yet it was possible for the material to sustain continued straining without failure to over double this value, that is, 33%.

We propose that it is not the twins themselves but the lack of plastic relaxation mechanisms that cause failure. Crystal plasticity calculations have shown that these regions at the tips or DTW/grain boundary junctions develop complex stress states, requiring at least five independent slip systems to satisfy compatibility (that is, the Taylor criterion)^45^. In most materials studied thus far, <c+a> is substantially harder to activate than basal <a> or prismatic <a> slip. In pure Mg, the ratio of stress thresholds for basal to pyramidal slip is 20–40 (refs 1, 46, 47), in AZ31, it is 12–15 (refs 3, 22), and in ZK60 it is 12 (ref. 48). Thus, the situation could arise that formation of free surface, for example, via local void formation or cracking, is more favourable than plastic relaxation. In HCP Mg–Li alloys, the ratio is smaller, being 8–10 (refs 11, 23). With the stress or energy required to activate pyramidal <c+a> slip closer to that for <a> slip and twinning, pyramidal <c+a> slip can offer another viable and distinct slip mode to satisfy this criterion, as has been shown in TEM-based studies^24^,^25^,^26^, and postpone local material separation.

To evaluate the plausibility of this hypothesis critically, we performed IGMA analyses and EPSC simulations (Supplementary Fig. 11). Supplementary Fig. 11a exhibits the IGMA distributions of DTWs and DTW containing grains, which reveal <uvt0> type distributions. According to Chun and Davies^49^ such IGMA distributions can result from the dominant activation of basal <a> slip or coactivation of basal <a> slip and pyramidal <c+a> slip. This analysis and the inverse pole figures given in Supplementary Figs 4 and 5 indicate that a high Schmid factor for pyramidal <c+a> slip evolves at strains > 10% suggesting that this slip mode is geometrically favoured, supporting the proposed role of this mode in plastic strain relaxation. Although geometric or similarly Schmid factor analyses can provide one form of assessing slip activity, they are not able to assess local grain-level effects. More specifically, grains, particularly in HCP metals like Mg alloys, have different stress states and strain states as a result of interactions with surroundings and their orientation. One way to assess local grain-level slip activity is via polycrystal, crystal plasticity-based modelling. Here, we employ EPSC polycrystal modelling, which accounts for elastic anisotropy as well as hardening via dislocation evolution according to thermally activate rate law^11^,^12^,^50^. With EPSC calculations of the deformation response (Supplementary Fig. 11b), we predict that basal <a> and pyramidal <c+a> slip are the dominant deformation modes at compression strains >10% being in good agreement with the IGMA analysis. Taken together, the combination of the IGMA, Schmid factor and EPSC analyses provide concurrent evidence of an important activation of <c+a> pyramidal slip, which has been observed directly in Mg–Li alloys using TEM^24^,^25^,^26^, can play a vital role in plastic strain relaxation within the vicinity of DTWs.

In summary, we take advantage of the sensitivity of 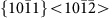 CTW to grain size to study the effect of 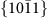
—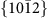 DTW on the compression failure strains and fracture of Mg-4wt%Li. For the same stress, strain, and loading path, the FG alloy (5 μm average grain size) developed a negligible fraction of DTWs, whereas the CG alloy (23 μm average grain size) developed a profuse fraction of DTWs. Yet, remarkably, we observe that both materials could be extended to large compression failure strains (> 30%). Thus, in contrast to prior implications made based on other Mg alloys, it is not necessary to eliminate DTW-ing to achieve high ductility. We rationalize that enhanced <c+a> slip in Mg-4wt%Li, relative to that in other common Mg alloys, provided an essential mechanism for plastic relaxation of the localized stresses generated by the thin DTWs. In fact, as demonstrated here, copious DTW-ing leads to 3D intra-granular networks that can reduce the effective grain size, a potential strengthening mechanism. Efforts to suppress twinning for the sake of increasing ductility would lose the opportunity for the strengthening effect provided by 3D twin networks and its possible stability against detwinning. This work provides key insight into effective designing of simultaneously strong and formable Mg alloys.

## Methods

### Microstructure and texture characterization

The deformed samples were investigated using optical microscopy and EBSD. Therefore, compression samples were cut in the centre and their cross-sections were prepared by standard metallographic methods including grinding, mechanical and chemical polishing. Chemical polishing was carried out using a solution of 12 ml hydrochloric acid (37%), 8 ml nitric acid (65%) and 100 ml ethanol. The EBSD analysis was performed using a Zeiss Ultraplus scanning electron microscope equipped with an Oxford Instruments EBSD detector NordlysNano. To enable the investigation of the samples using optical microscopy the samples were etched subsequent to chemical polishing using a solution of 5 g citric acid and 95 ml distilled water. The grain size of the extrusions was obtained from optical micrographs using the software ImageJ^51^. The texture of the as-extruded material was determined using laboratory X-ray diffraction (Co-K_α_); the orientation distribution functions were calculated using the MTEX software package^52^.

### Mechanical testing

The compression tests were conducted using a universal testing machine MTS 810 and a strain rate of 3.3 × 10^−4^ s^−1^. The cylindric compression die is made of an Inconel alloy and no additional lubrication was applied. The barrelling of the sample is shown schematically in Supplementary Fig. 2.

## Additional information

**How to cite this article:** Lentz, M. *et al*. Strength and ductility with 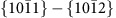 double twinning in a magnesium alloy. *Nat. Commun.* 7:11068 doi: 10.1038/ncomms11068 (2016).

## Supplementary Material

Supplementary Information Supplementary Figures 1-11, Supplementary Table 1 and Supplementary Notes 1-2
