# Supplementary material for: Strength and ductility with {101̄1} — {101̄2} double twinning in a magnesium alloy
Source: Nat Commun. 2016 Apr 4;7:11068. doi: 10.1038/ncomms11068 (PMC5482718; doi:10.1038/ncomms11068)
Supplement: Supplementary Information — Supplementary Figures 1-11, Supplementary Table 1 and Supplementary Notes 1-2 [file ncomms11068-s1.pdf]

Supplementary Information

Supplementary Figures

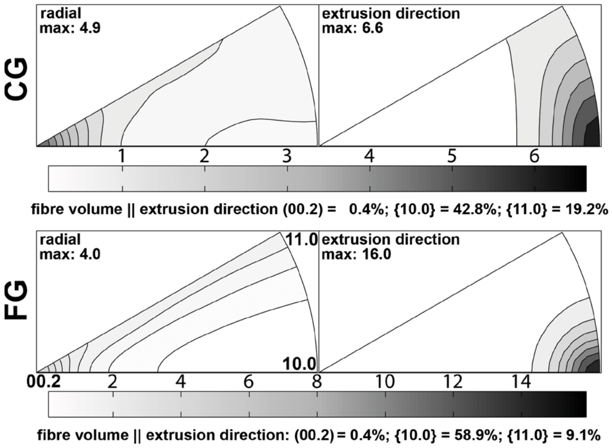

Supplementary Figure 1 | Inverse pole figures representing the as-extruded texture parallel to radial and extrusion direction. The textures of both materials are qualitatively similar, but differ in sharpness. Calculating the fiber volume fraction within 15° about the extrusion direction confirms a strengthened <10.0> texture component parallel to ED in the FG material.

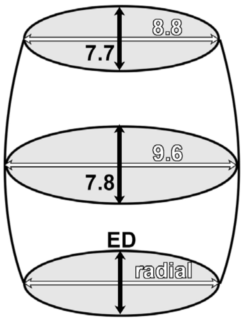

Supplementary Figure 2 | Exemplary schematic of the barreling of a compression sample at 29.5% strain. Non-uniform deformation featuring a more pronounced lateral extension perpendicular to the ED direction. This non-uniform deformation is introduced by the as-extruded texture and the selected strain path, while the barreling of the samples is minor.

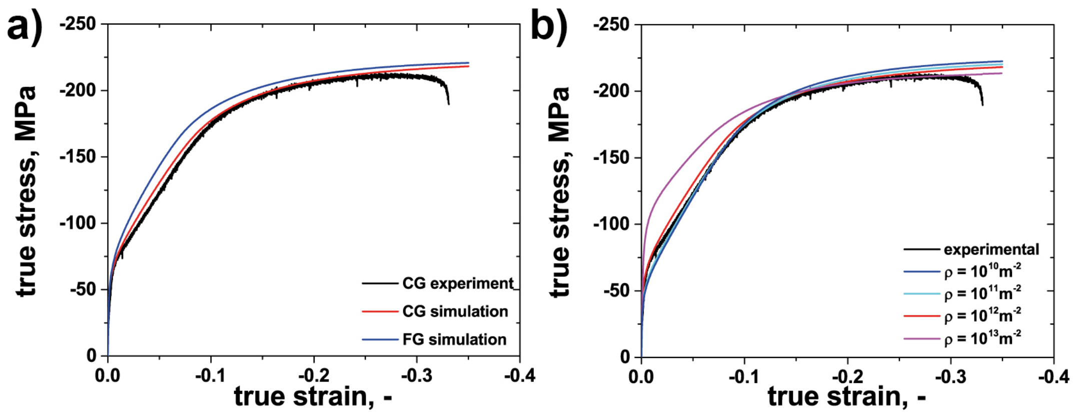

Supplementary Figure 3 | EPSC simulations displaying the effect of the different starting textures and dislocation densities. a) The difference in texture results in minor changes of the flow curve. b) The impact of different dislocation densities is negligible considering low dislocation densities, which are reasonably assumed based on the recrystallized microstructure of both materials.

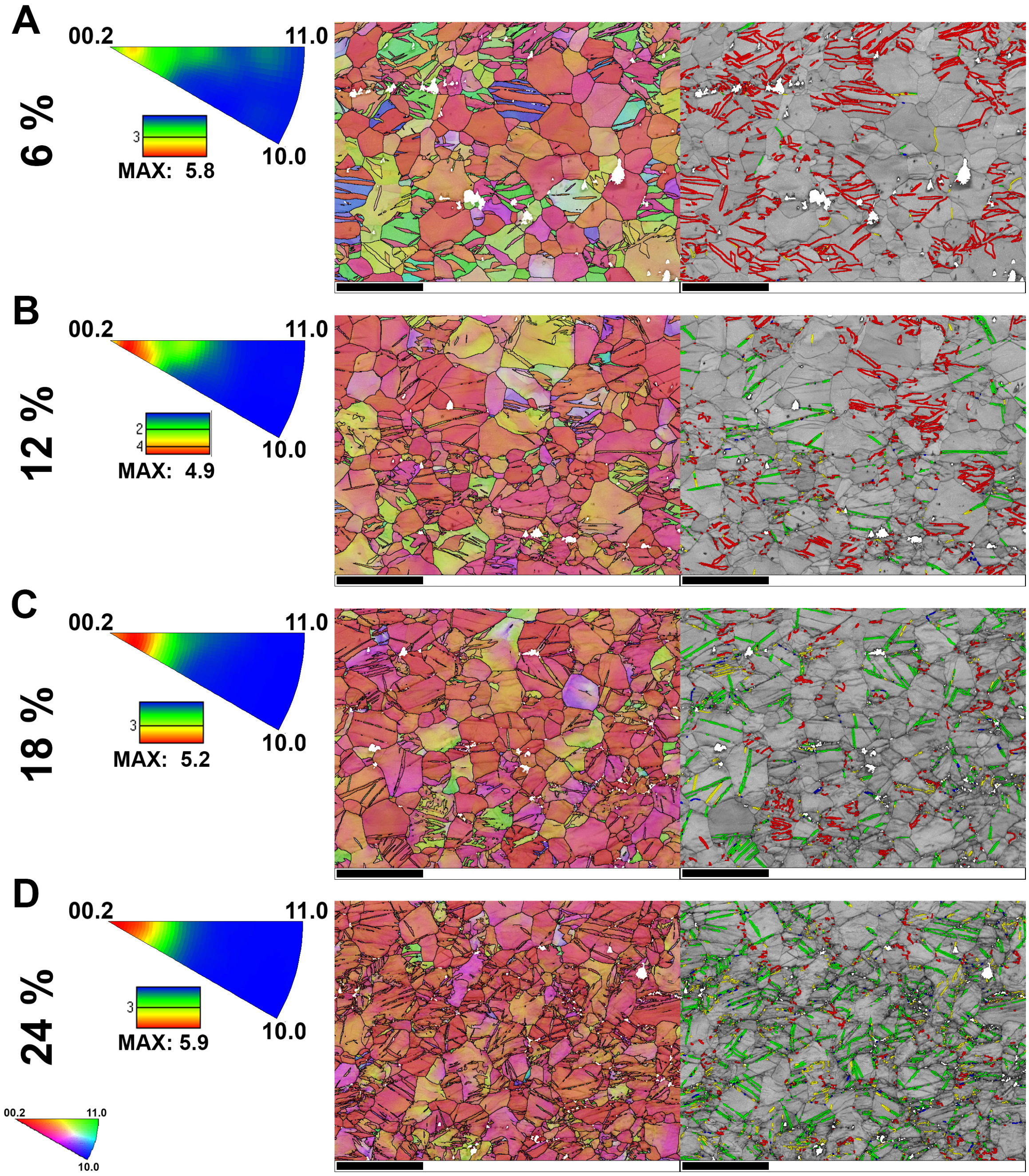

Supplementary Figure 4 | EBSD analysis of the coarse-grained material. EBSD analysis using inverse pole figures (IPF), IPF maps superimposed by band contrast (BC) and twin boundaries (TB) superimposed by (BC) maps of CG samples for different strain levels. TTW boundaries = red, CTW = yellow, type 1 DTW = green, type 2 DTW = blue. The scale bars correspond to 100 μm.

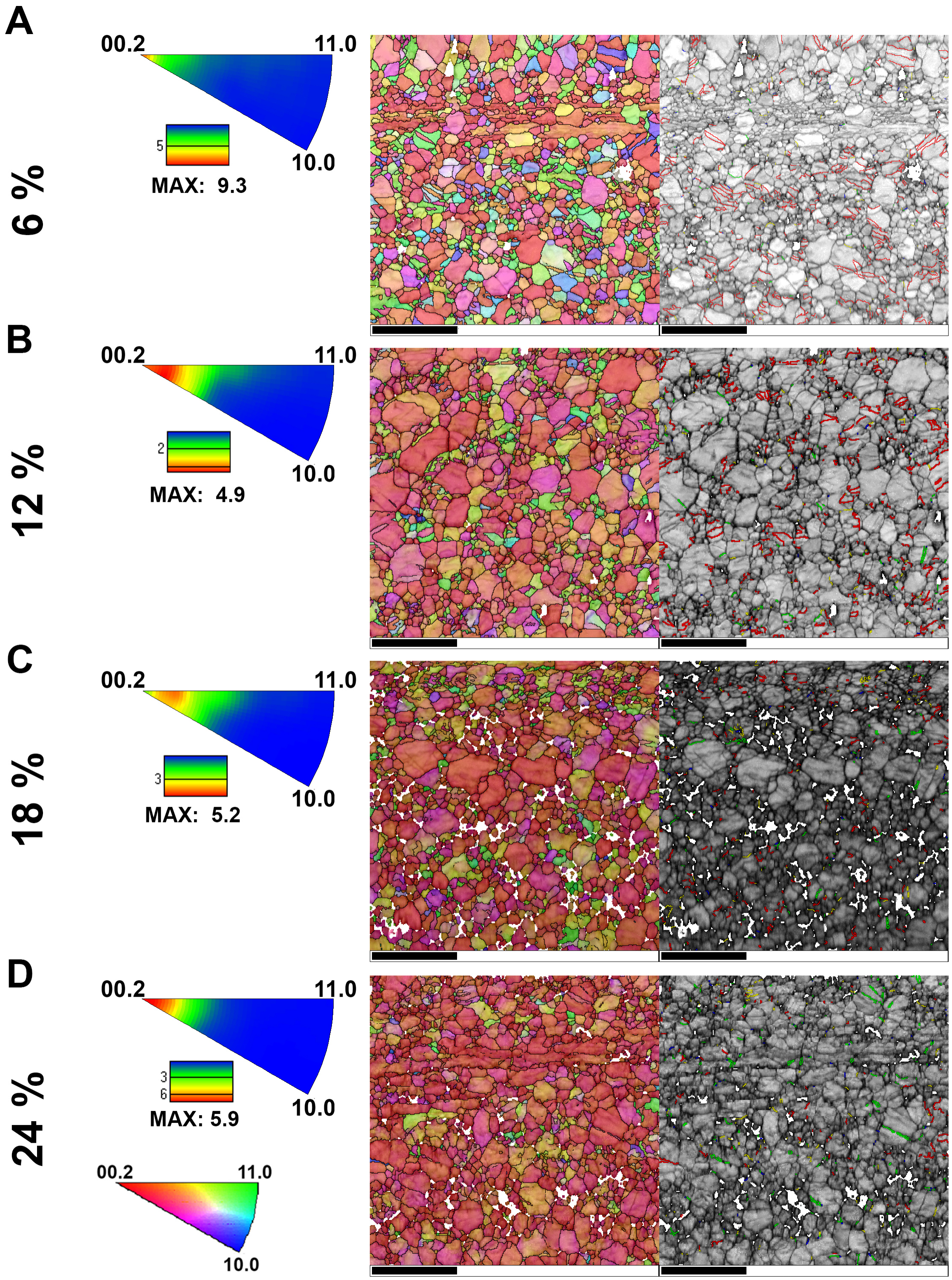

Supplementary Figure 5 | EBSD analysis of the fine-grained material. EBSD analysis using inverse pole figures (IPF), IPF maps superimposed by band contrast (BC) and twin boundaries (TB) superimposed by (BC) maps of FG samples for different strain levels. TTW boundaries = red, CTW = yellow, type 1 DTW = green, type 2 DTW = blue. The scale bars correspond to 50 µm.

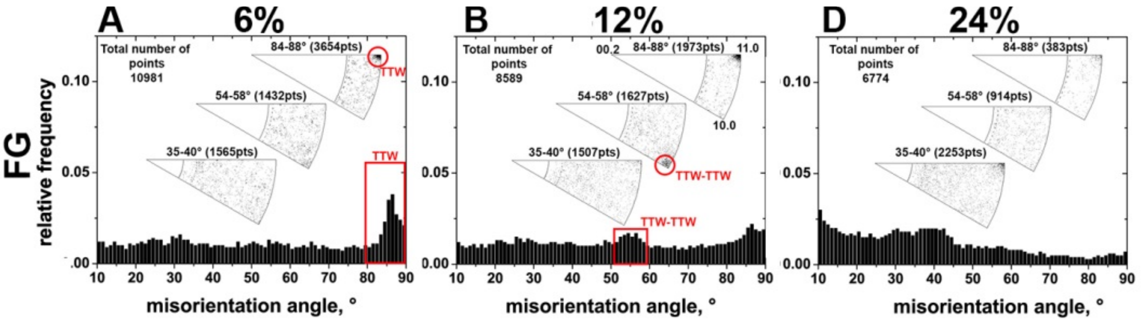

Supplementary Figure 6 | Misorientation distribution functions of the FG material at different strains. At ε_E = 6 % TTW-ing is predominant, although it is hindered in comparison to the CG material. DTWs were observed only very occasionally within the investigated strain range. The very weak peak at ~ 55° can be attributed to TTW-TTW intersections.

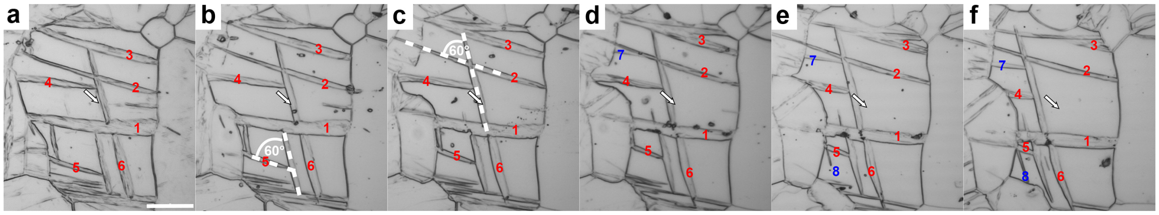

Supplementary Figure 7 | Slices illustrating the 3D arrangement of several contraction twins. The white arrow is used as a reference point facilitating the tracking of twin boundary shifts. Several contraction twins are labeled with red numbers to facilitate the relocation of the twins in each of the sections. In addition blue numbers label contraction twins, which were not visible initially and appeared during sectioning. The scale bar corresponds to 20 µm.

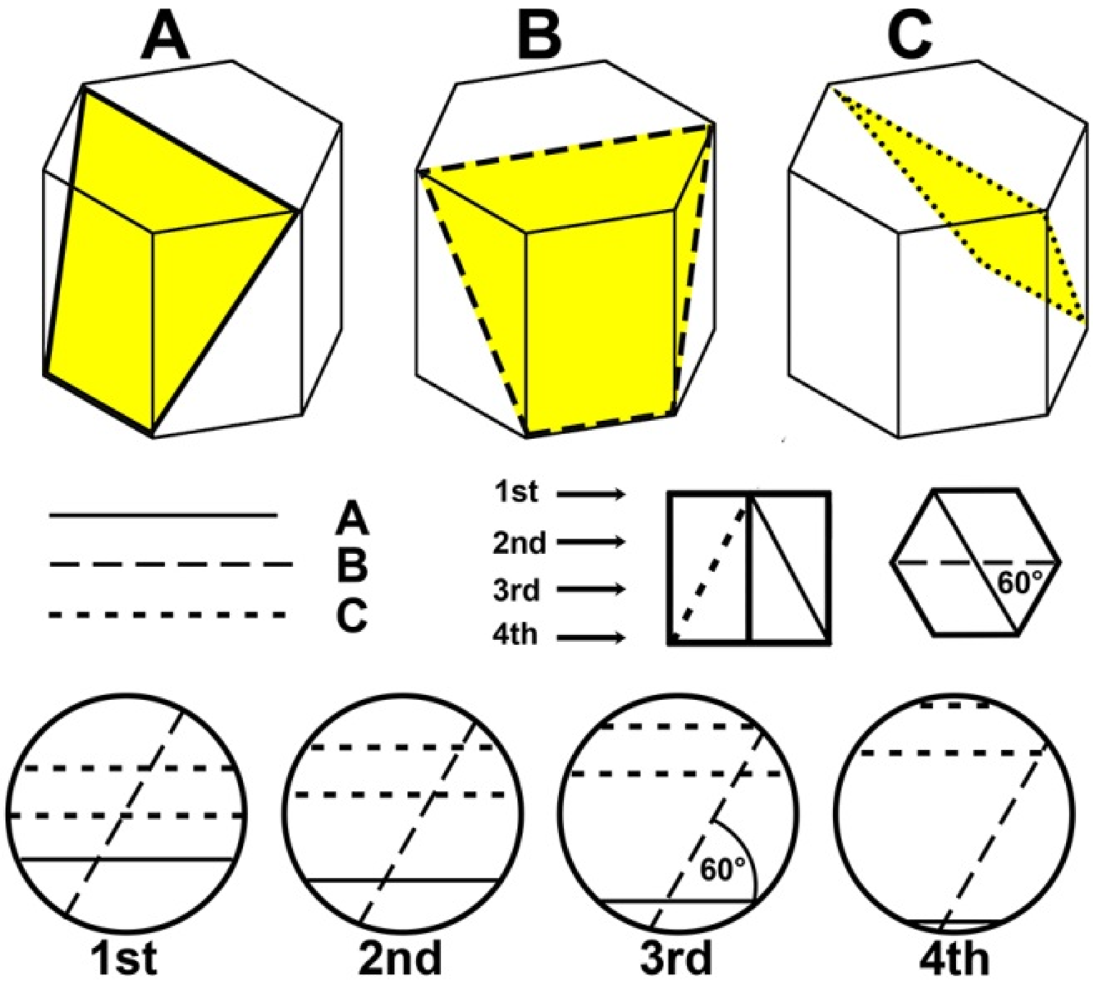

Supplementary Figure 8 | Schematic displaying contraction twin networks. Schematic illustrating the 3D network given in Fig 4 a-c and Supplementary Fig. 7. Twin 1 corresponds to A, twin 6 corresponds to B and twins 2, 3, 5 correspond to C. Twins A and C share a <11.0> zone axis and hence appear to be parallel in each section; however, sectioning reveals different variants through the increasing distance between the twins. Twin B does not share a <11.0> zone axis with the twins A and C. This twin variant is rotated by 60° about a <00.2> axis with respect to twins A and C. This 60° angle is maintained through serial sectioning.

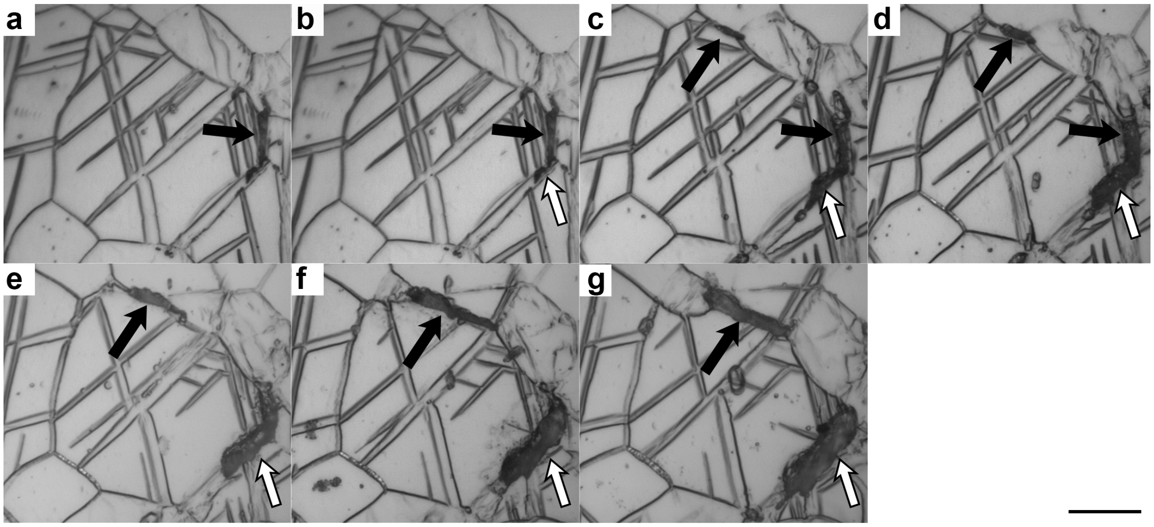

Supplementary Figure 9 | Slices displaying crack formation. Slices illustrating crack formation at grain boundaries (black arrows) and at contraction twin boundaries (white arrows). The scale bar corresponds to 20 µm.

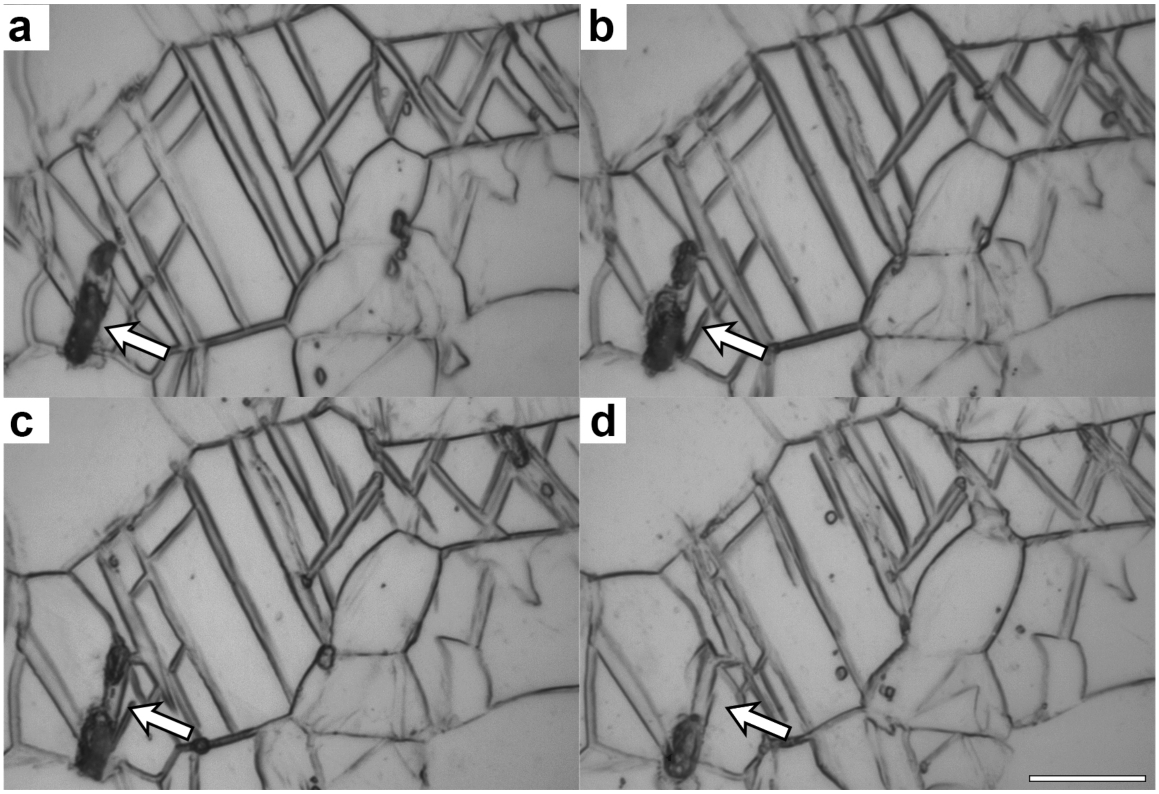

Supplementary Figure 10 | Slices illustrating crack formation at contraction twin boundaries. White arrows mark a crack, which was formed at a contraction twin boundary using consecutive slices. The scale bar corresponds to 20 μm.

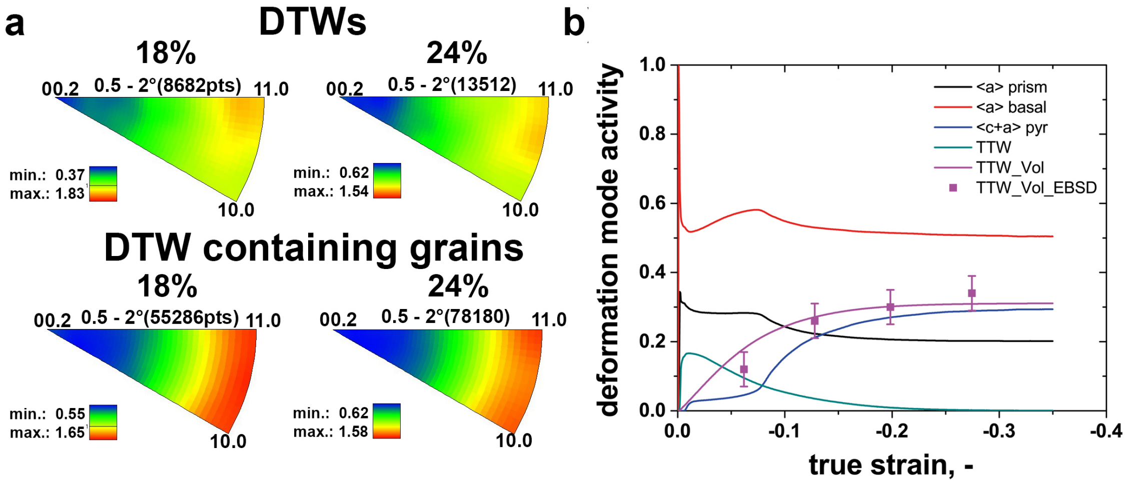

Supplementary Figure 11 | Analysis of the active deformation modes. a) IGMA distributions of the DTWs and DTW containing grains. The IGMA distributions are of <uvt0> type indicating a predominant activation of basal <a> slip or coactivation of basal <a> slip and pyramidal <c+a> slip. b) Predicted deformation mode activity for the CG material indicating an important contribution of <c+a> pyramidal slip at strains > 10%.

Supplementary Tables

Supplementary Table 1 | Tension twin (TTW) volume fractions of the CG and the FG materials. The TTW volume fractions have been calculated from the <00.2> texture component parallel to the compression direction (cf. Supplementary Notes - TTW volume fraction)

Compression strain	TTW volume fraction (CG)	TTW volume fraction (FG)
ε_E = 6%	12% ± 5%	12% ± 5%
ε_E = 12%	26% ± 5%	25% ± 5%
ε_E = 18%	30% ± 5%	24% ± 5%

Supplementary Note 1: Texture

Fig. 1 displays the initial texture of the extrusion using pole figures and inverse pole figures (IPF) with respect to the radial direction (⊥ ED). In order to provide a more detailed description of the texture Supplementary Fig. 1 displays IPFs parallel to the extrusion and the radial direction along with the fibre volume fraction of the relevant texture components. Both materials feature very similar textures, which only differ slightly in sharpness. Hence, texture effects are largely omitted in this study. In order to evaluate the remaining texture effects, elastic-plastic self-consistent simulations were conducted, which are displayed in Supplementary Fig. 3 a) and confirm a minor influence of the starting texture on the deformation behavior.

Supplementary Note 2: Tension twin volume fraction

We calculate the fiber volume of the <0002> texture component parallel to compression direction within an angular deviation of 30° and subtracting the initially present fiber volume. The results are summarized in Supplementary Table 1. Unfortunately, the determination of the initially present fiber volume introduces a notable error, which might conceal the commonly observed Hall-Petch effect on tension twinning.
